# Exploring the association between functional gains and length of stay for stroke patients in 2 inpatient rehabilitation facilities in Riyadh city: A retrospective cohort study

**DOI:** 10.1097/MD.0000000000050623

**Published:** 2026-09-11

**Authors:** Bashayer A. AlHarbi, Faisl AlGarni, Taalaf AlWaqid, Mastourah AlShahrani, Taibah AlShanqiti, Aqeel M. Alenazi, Norah A. Alhwoaimel, Bader Alqahtani

**Affiliations:** a Saudi Medical Appointments and Referrals Center, Ministry of Health, Riyadh, Saudi Arabia; b Erada & Mental Health Hospital, AlKharj, Riyadh, Saudi Arabia; c Department of Health and Rehabilitation Sciences, College of Applied Medical Sciences, Prince Sattam Bin Abdulaziz University, Al-Kharj, Riyadh, Saudi Arabia; d Asir Central Hospital, Abha, Asir, Saudi Arabia; e Home Healthcare Department, Madinah, Saudi Arabia.

**Keywords:** functional gain, length of stay, Saudi Arabia, Stroke

## Abstract

Stroke remains an important healthcare issue globally, as it causes substantial morbidity and mortality in the population. The relationship between length of stay (LOS) and the functional outcomes of stroke rehabilitation has become an increasingly important area of investigation. Substantial research supports the idea that an extended rehabilitation stay is associated with enhanced functional recovery. This study aimed to explore the relationship between functional recovery, measured using the functional independence measure (FIM), and the LOS, and to analyze the possible predictability of functional recovery by the LOS for stroke patients who underwent an intensive rehabilitation program at the inpatient rehabilitation facility. This retrospective cohort study utilized existing data from the medical records of stroke patients who had undergone an intensive rehabilitation program at 2 tertiary centers in Riyadh city with an inpatient rehabilitation facility. Multivariate linear regression analysis was used to explore the association between FIM scores and LOS. The total included sample was subdivided according to LOS to explore the association between the FIM and other sample demographics and clinical characteristics. A total sample size of 400 was divided into 3 groups: short LOS (≤ 30 days) (n = 134), moderate LOS (31–60 days) (n = 234), and long LOS (> 61 days) (n = 32), with mean standard deviation age of 60.3 ± 14.3, 59.9 ± 13.8, and 60.1 ± 15.6 years, respectively. Total-FIM and motor-FIM gains were significantly lower in the short LOS group than in the long LOS group, while motor-FIM gain was also significantly lower in the moderate LOS group. No significant association was found between LOS groups and cognitive-FIM gain. Rehabilitation programs involved diverse interventions delivered through all rehabilitation therapies. Patients with longer rehabilitation duration achieved significantly greater total-FIM and motor-FIM gains, particularly compared with those in the short LOS group. Moderate LOS was also associated with lower motor-FIM gain than long LOS. In contrast, cognitive-FIM gain was not significantly associated with LOS, suggesting that overall and motor recovery may benefit more consistently from longer inpatient rehabilitation duration.

## 1. Introduction

Stroke remains an important healthcare issue in Saudi Arabia, as it causes substantial morbidity and mortality in the population. Current research indicates an increasing burden of stroke, with worrying figures regarding incidence and prevalence trends. The incidence of stroke is predicted to increase from 16,553 in year 1 to 27,626 in year 10, representing an increase of 67%. This increase places an enormous burden on healthcare resources.^[1,2]^

Stroke rehabilitation is a critical phase in the continuum of care and significantly influences a patient’s ability to regain independence and quality of life. Among the various indicators used to assess the effectiveness of rehabilitation, the functional independence measure (FIM) is widely adopted, reflecting both motor and cognitive improvements. Concurrently, length of stay (LOS) in inpatient rehabilitation has emerged as a practical and clinical metric linked to recovery outcomes. Understanding how LOS relates to functional gains is essential for optimizing rehabilitation planning, resource allocation, and individualized care delivery, particularly in countries such as Saudi Arabia, where the incidence of stroke is increasing across both elderly and middle-aged populations. A growing body of literature suggests that an extended LOS may support better functional recovery. However, a more in-depth analysis of the pure association between functional gains and LOS, and the interaction of this association with considerable covariates from demographic characteristics such as age and sex; stroke-related factors such as severity, type, location, and the affected body parts; comorbidities; and number of medications, will add comprehensive informative evidence to the literature. Moreover, the rehabilitation interventions provided during the inpatient stay, which were not directly evaluated in terms of causal impact in this study, play a fundamental role in recovery and are essential for documentation and review. An overview of therapy types, session intensity, and intervention focus can provide contextual insights into the therapeutic environment experienced by patients across different LOS groups. This study aimed to explore the association between functional gains and LOS among stroke patients in 2 inpatient rehabilitation facilities in Riyadh. In parallel, it reviews the rehabilitation program structure and profiles the stroke-related, demographic, and socioeconomic characteristics of the study cohort to understand how these factors reflect LOS and recovery outcomes. Through this comprehensive approach, this study seeks to contribute data-driven insights that support evidence-based rehabilitation practices and planning.

There is a need to understand the association between inpatient rehabilitation duration (defined as LOS in this study) and functional recovery in patients with stroke. In addition, there is a need to consider how rehabilitation programs, stroke-related characteristics, demographics, and other factors are reflected in both LOS in inpatient rehabilitation facilities and functional gain. Therefore, this study primarily aimed to explore the relationship between functional recovery, measured by FIM, and LOS, and to analyze the possible predictability of functional recovery by LOS for stroke patients who underwent an intensive rehabilitation program at the inpatient rehabilitation facility (IRF). Secondarily, we reviewed the stroke-related characteristics, rehabilitation program details, and other considerable variables having a covariance effect on both stroke functional recovery outcomes and LOS demonstrated in the literature.

## 2. Methods

### 2.1. Study design and data collection

**Figure 1.**
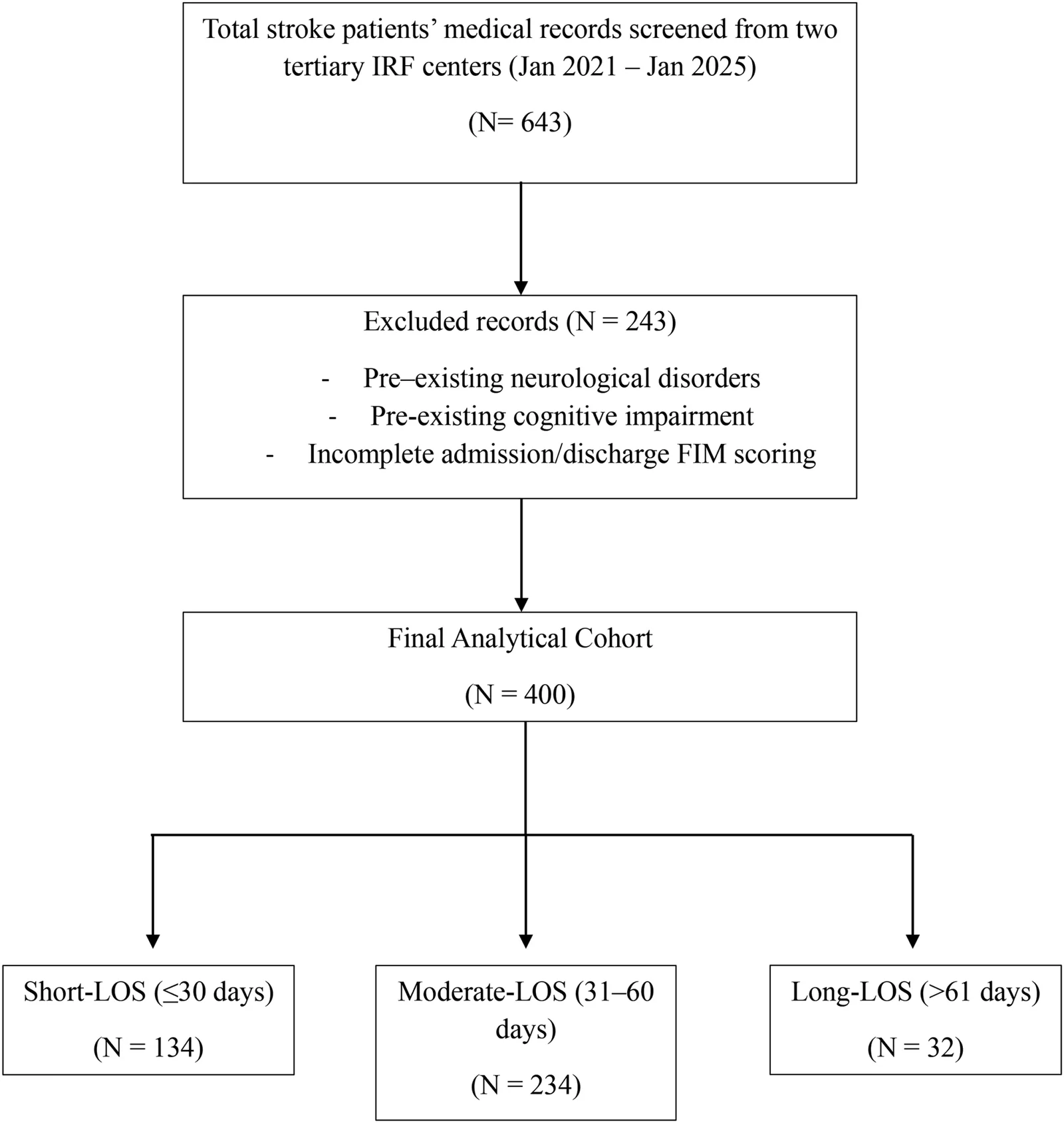
Study participants flowchart. FIM = functional independence measure, IRF = inpatient rehabilitation facility, LOS = length of stay, N = number of records.

A retrospective cohort study was conducted by extracting existing data from the medical records of stroke patients who had undergone an inpatient rehabilitation program in 2 main tertiary medical cities with an IRF in Riyadh city between 2022–2025. The first was Sultan Bin Abdulaziz Humanitarian City, and the second was King Fahad Medical City, with a total of 400 patient medical records included (Fig. 1). The available data from the electronic databases of the 2 centers outlined the timeframe of the collected data. An electronic form was designed for study investigators to collect the study variables, including all independent variables, dependent variables, and covariates. The form consisted of items with mandates to obtain the demographic, clinical, and socioeconomic characteristics of the sample. In addition, detailed information regarding the rehabilitation program, such as subspecialties, treatment plans, treatment goals, and functional outcome measures, was used at admission and discharge from inpatient rehabilitation.

### 2.2. Study sample and enrollment of reviewed medical records

Participants’ medical records were included if they were aged 18 years, had ischemic or hemorrhagic stroke according to the patients’ needs or the stroke recovery individualized inpatient rehabilitation program, and had information in their medical records regarding the rehabilitation program, including, but not limited to, FIM scores at admission and discharge from the IRF. Exclusion criteria were applied to any patient with a medical record of a preexisting neurological disorder, preexisting severe cognitive impairment, or incomplete scoring of FIM either at admission or at discharge.

### 2.3. Study outcome measures

The first outcome measure was the FIM, a rehabilitation outcome measure used for a prolonged period, with efficient evidence of its effectiveness in measuring functional physical and cognitive abilities. It consists of 18 items, 13 for the physical domain and 5 for the cognitive domain, with a rating scale for each item from 1 to 7, with 1 indicating the least ability that represents total dependence on others and 7 the largest ability score that highlights maximum normal independence, with a total score of 126 for total FIM, 91 for motor FIM, and 35 for cognitive FIM. The second outcome measure in this study was the LOS in the IRF, measured as the difference between admission and discharge dates to the IRF.

### 2.4. Study variables

The independent variable was LOS, while the dependent variable was FIM, subcategorized into total FIM, motor FIM, and cognitive FIM. Other variables considered as covariates according to the literature review included demographic, socioeconomic, and clinical characteristics, including stroke factors (severity, type, location, body involvement represented by the type of body paralysis, and stroke recurrence) and rehabilitation program characteristics.

### 2.5. Ethical consideration

Ethics Approval was obtained from the Ethics Committee of the Department of Health and Rehabilitation Sciences at Prince Sattam Bin Abdulaziz University approval number (Instituitional Review Board No. RHPT/024/014) to ensure compliance with the ethical standards related to research on human subjects. Key ethical considerations include the confidentiality of patient information obtained from medical records and ensuring that patient data are anonymized by coding each medical record during data collection.

### 2.6. Data analysis

The data collection form was transformed into an Excel sheet to arrange and organize the data in the analytical tables. The sample was stratified into 3 groups according to LOS to further analyze the association between FIM total and subcategories with LOS levels defined in this study as short, moderate, and long LOS. Descriptive statistics with means and standard deviations for the sample demographics and clinical characteristics were used to compare the means between LOS groups. Chi-square tests were conducted for categorical variables such as gender and stroke type. Multivariate linear regression analysis was used to assess the association between LOS and FIM scores. An analysis of the rehabilitation intervention categories across a total sample of 400 patients was conducted to determine the distribution and frequency of the therapies employed during inpatient rehabilitation. The interventions were classified into thematic categories based on therapeutic intent and modality. Statistical Package for the Social Sciences 26 and STATA-12 (StataCorp)were used for statistical analyses.

## 3. Results

As shown in Table 1, the average LOS was 23.8 days for the short-stay group (≤ 30 days), 43.5 days for the moderate-stay group (31–60 days), and 90.4 days for the long-stay group (> 61 days). No statistically significant differences were found in age between groups, with the mean age ranging from 59.9 to 60.3 years (*P* = .972). Sex distribution was similar across groups (*P* = .851), with women making up the majority of participants in each category. The stroke types were almost equal in all groups, with no significant differences in the distribution of both stroke types across the 3 groups (*P* = .665). Higher percentages of both ischemic and hemorrhagic strokes were observed in the moderate-stay group: 60.5% for ischemic stroke and 55.6% for hemorrhagic stroke, and 31.8% and 34.9% in the short-stay group for ischemic and hemorrhagic strokes, respectively. In the long-stay group, 7.6% of the total sample had ischemic stroke and 9.4% had hemorrhagic stroke. Regarding stroke location, significant differences between the locations of stroke were observed among the entire sample (*P* = .012), and the number of strokes affecting the left hemisphere was superior to those affecting the right and bilateral hemispheres. The left hemisphere strokes affected the short-stay (31.8%) and moderate-stay groups (64.1%) more than the long-stay group (4%). The right hemisphere was affected in the short-stay (36.8%) and moderate-stay (50.5%) groups more than the long-stay (4.3 %) groups. Stroke affecting the bilateral hemispheres was also observed in 9 patients in all groups: (22.2%) were in the short-stay group, (66.6%) were in the moderate-stay group, and the remaining (11.1%) were in the long-stay group. All groups demonstrated functional improvement between admission and discharge, as measured by the total-FIM, motor-FIM, and cognitive-FIM gains. The long-stay group showed the highest mean total-FIM gain (25.9 ± 14.3), followed by the moderate-stay group (19.1 ± 12.7) and the short-stay group (11.7 ± 10) (*P* < .0001). Motor-FIM gain followed a similar trend, with significantly greater improvements in the long-stay group (21.6 ± 11.4) compared to the moderate-stay group (15.7 ± 11.7) and short-stay group (9.9 ± 9.2) (*P* < .0001). Cognitive-FIM gain also increased with longer LOS, with values of 1.8 ± 2.6, 3.4 ± 4.3, and 4.2 ± 8.1 for short, intermediate, and long groups, respectively (*P* < .0001).

**Table 1:** Demographic and clinical characteristics of study sample (N = 400).

Variables	LOS ≤ 30 d N = 134	LOS, 31–60 d N = 234	LOS > 61 d N = 32	*P*
Age in yrs, mean ± SD	60.3 ± 14.3	59.9 ± 13.8	60.1 ± 15.6	.972
Gender, N (%)				.851
Men	41 (32.5)	76 (60.3)	9 (7.1)	
Women	93 (33.9)	158 (57.6)	23 (8.3)	
Stroke type, N (%)				.665
Ischemic	83 (31.8)	158 (60.5)	20 (7.6)	
Hemorrhagic	37 (34.9)	59 (55.6)	10 (9.4)	
Stroke location, N (%)				.012
Left hemisphere	63 (31.8)	127 (64.1)	8 (4)	
Right hemisphere	67 (36.8)	92 (50.5)	23 (4.3)	
Bilateral	2 (22.2)	6 (66.6)	1 (11.1)	
FIM, mean ± SD				
Total-FIM gain	11.7 ± 10	19.1 ± 12.7	25.9 ± 14.3	< .0001
Motor-FIM gain	9.9 ± 9.2	15.7 ± 11.7	21.6 ± 11.4	< .0001
Cognitive-FIM gain	1.8 ± 2.6	3.4 ± 4.3	4.2 ± 8.1	< .0001
LOS, mean ± SD	23.8 ± 5.8	43.5 ± 7.9	90.4.±29.9	< .0001

FIM = functional independence measure, LOS = length of stay, N = number of records, SD = standard deviation.

Multivariable linear regression analysis in Table 2 was conducted to examine differences in FIM gain across LOS categories, using the long LOS group (≥ 61 days) as the reference category. In the unadjusted model, patients with a short LOS (0–30 days) demonstrated significantly lower total-FIM gain compared with those with a long LOS (*β* = −13.39, 95% CI: −19.56–−7.23; *P* < .001), while the difference between the moderate LOS group (31–60 days) and the long LOS group was not statistically significant (*β* = −4.96, 95% CI: −11.08–1.16; *P* = .112). A similar pattern was observed for motor-FIM gain, where both the short LOS group (*β* = −12.10, 95% CI: −16.68–−7.52; *P* < .001) and the moderate LOS group (*β* = −5.46, 95% CI: −10.03–−0.88; *P* = .020) showed significantly lower gains than the long LOS group. In contrast, no significant differences were identified in cognitive-FIM gain for either the short LOS group (*β* = −1.29, 95% CI: −4.68–2.10; *P* = .455) or the moderate LOS group (*β* = 0.50, 95% CI: −2.79–3.78; *P* = .766). After adjustment for age, gender, and stroke location, the findings remained largely unchanged. Specifically, short LOS remained significantly associated with lower total-FIM gain (*β* = −13.66, 95% CI: −19.77–−7.54; *P* < .001) and lower motor-FIM gain (*β* = −12.32, 95% CI: −16.83–−7.82; *P* < .001), whereas the association with cognitive-FIM gain remained nonsignificant (*β* = −1.33, 95% CI: −4.69–2.02; *P* = .436). For the moderate LOS group, motor-FIM gain continued to be significantly lower than that of the long LOS group after adjustment (*β* = −5.97, 95% CI: −10.50–−1.45; *P* = .010), while differences in total-FIM gain (*β* = −5.52, 95% CI: −11.59–0.54; *P* = .074) and cognitive-FIM gain (*β* = 0.45, 95% CI: −2.80–3.69; *P* = .786) were not statistically significant. Overall, a longer LOS was associated with greater improvement in total and motor functional outcomes, whereas cognitive improvement did not differ significantly across LOS categories.

**Table 2:** Multivariable logistic regression estimates of LOS and FIM-based functional gains.

FIM scale	Model 1 (unadjusted)	Model 1 (unadjusted)	Model 2 (adjusted)	Model 2 (adjusted)
	* Short LOS vs‡ long LOS	† Moderate LOS vs‡ long LOS	* Short LOS Vs‡ long LOS	† Moderate LOS vs‡ long LOS
	*β* (95% CI); *P* value	*β* (95% CI); *P* value	*β* (95% CI); *P* value	*β* (95% CI); *P* value
Total-FIM gain	−13.39 (−19.56–−7.23); < .001	−4.96 (−11.08–1.16); .112	−13.66 (−19.77–−7.54); < .001	−5.52 (−11.59–0.54); .074
Motor-FIM gain	−12.10 (−16.68–−7.52); < .001	−5.46 (−10.03–−0.88); .020	−12.32 (−16.83–−7.82); < .001	−5.97 (−10.50–−1.45); .010
Cognitive-FIM gain	−1.29 (−4.68–2.10); .455	0.50 (−2.79–3.78); .766	−1.33 (−4.69–2.02); .436	0.45 (−2.80–3.69); .786

Reference: long LOS.

Model 1: unadjusted covariates (age, gender and stroke location).

Model 2: adjusted covariates (age, gender and stroke location).

Bolded results: significant at *P* < .05.

CI = confidence interval, FIM = functional independence measure, LOS = length of stay.

* Short LOS: 0 to 30 days.

† Moderate LOS: 31 to 60 days.

‡ Long LOS: ≥ 61 days.

## 4. Discussion

This study has several important clinical and policy implications. First, given the rising healthcare costs and resource constraints, this study adds empirical support to the ongoing discussions on the ideal length of rehabilitation for stroke patients. This study offers a comprehensive understanding of how various aspects of recovery (such as motor vs cognitive) react to rehabilitation intensity and duration by differentiating the effects of short, moderate, and extended rehabilitation durations on specific FIM domains. Second, the results directly affect inpatient rehabilitation facilities’ (IRFs) resource allocation, discharge procedures, and rehabilitation planning. Healthcare administrators and legislators may utilize these data to create evidence-based standards for stroke rehabilitation programs and enhance care quality and efficiency if LOS is demonstrated to be a valid predictor of functional gain. Third, this study addressed the pressing need for context-specific rehabilitation guidelines in Saudi Arabia, where lifestyle changes and demographic trends have resulted in a marked increase in the incidence of stroke among younger age groups. The findings of this study can inform national initiatives to improve recovery outcomes and poststroke care and eventually reduce the long-term burden of disability. Ultimately, the study methodology, which combines robust multivariate regression modeling with retrospective data analysis, provides a repeatable framework for further research, and the results could be used as a basis for developing prospective studies and intervention trials targeted at improving rehabilitation protocols. A total of 400 stroke patients were included in the analysis, stratified into 3 groups based on rehabilitation LOS: short-stay LOS ≤ 30 days (n = 134), moderate-stay LOS 31 to 60 days (n = 234), and long-stay LOS ≥ 62 days (n = 32). The mean age was approximately 60 years in each LOS category (60.3 ± 14.3, 59.9 ± 13.8, and 60.1 ± 15.6 years for the short-, moderate-, and long-stay groups, respectively), with no significant difference between groups (*P* = .972). This is consistent with previous studies that reported a similar median age for their patient groups (60.0 ± 14.6).^[3]^ Additionally, a median age of 61 years was reported in a study conducted at a subacute rehabilitation facility in South Africa.^[4]^ In parallel with the literature, and compared to past years, there is an increase in the incidence of stroke in younger populations aged 45 years and above.^[1,2]^ Sex distribution was also comparable across the LOS groups. Overall, females constituted approximately 68.5% of the sample (274 women vs 126 men), which aligned with studies that showed a trend toward a higher number of females in their samples.^[4,5]^ However, this finding is inconsistent because of differences between our findings and those reported in similar studies in the literature. Regarding stroke type, the majority of patients had ischemic strokes (approximately two-thirds of the cohort) versus hemorrhagic strokes (approximately one-third), and these proportions did not significantly differ between LOS groups (*P* = .665). Similarly, the ischemic type was more common than the hemorrhagic type among the samples,^[3–5]^ which is also supported by the literature and clinical judgment that the most common type of stroke is ischemic in most populations. The difference in stroke location was significant between the right and left hemispheres (*P* = .012). The left hemisphere was more affected than the right hemisphere in all LOS groups. Between-group comparison also demonstrated a difference, with the left hemisphere being the most affected side, similar to Bijl et al (2023), who found a higher incidence of left hemisphere stroke in 51.4% of their sample.^[4]^ However, differences in findings have been observed in some studies. Bindawas et al (2018) showed that 48.4% of their sample had a stroke in the right hemisphere, and 34.4% had a stroke in the left hemisphere.^[3]^

The present regression analysis demonstrated that LOS was significantly associated with functional improvement following inpatient stroke rehabilitation, particularly for total-FIM and motor-FIM gain. Compared with patients with a long LOS (≥ 61 days), those with a short LOS (0–30 days) had significantly lower total-FIM and motor-FIM gains in both unadjusted and adjusted models, while patients with a moderate LOS (31–60 days) also showed lower motor-FIM gain. In contrast, cognitive-FIM gain did not differ significantly across LOS categories, even after adjustment for age, gender, and stroke location. These findings suggest that a longer rehabilitation stay may contribute more strongly to physical and overall functional recovery than to cognitive recovery. This pattern is broadly consistent with prior studies showing that longer inpatient rehabilitation is associated with greater functional gains after stroke. Bindawas et al (2018) reported that LOS was significantly related to functional outcomes among patients discharged from an IRF in Saudi Arabia, supporting the present finding that prolonged inpatient care is linked with better total functional improvement.^[3]^ Similarly, Camicia et al (2016) found that LOS in inpatient rehabilitation facilities was associated with stroke outcomes, while Tung et al (2021) specifically concluded that a longer post-acute care stay resulted in greater functional improvement in poststroke patients.^[5,6]^ Together, these studies reinforce the interpretation that extended rehabilitation exposure may provide greater opportunity for repetitive training, multidisciplinary intervention, and progressive recovery, particularly in motor domains.

The present results are also in agreement with evidence emphasizing the central role of motor recovery in poststroke rehabilitation outcomes. Alawieh et al (2018) highlighted that poststroke motor recovery is shaped by multiple biological and therapeutic factors, while Lin and Dionne (2018) and Shahid et al (2023) emphasized that rehabilitation interventions often target movement restoration and functional performance.^[7–9]^ In this context, the significantly lower motor-FIM gain observed in the short- and moderate LOS groups relative to the long LOS group may reflect insufficient time to achieve meaningful motor relearning and task-specific adaptation. This interpretation is further supported by Yagi et al (2017) and Fujino et al (2021), who underscored the importance of rehabilitation exposure and intensity in influencing recovery trajectories after stroke.^[10,11]^ A notable finding in the present study is that the association between LOS and functional recovery remained significant after adjustment for age, gender, and stroke location, particularly for total-FIM and motor-FIM gain in the short LOS group. This suggests that LOS may have an independent relationship with rehabilitation outcome beyond these demographic and clinical factors. Such a finding aligns with prior literature identifying LOS as an important determinant of outcome, while also recognizing that stroke recovery is multifactorial. Harvey (2015), Rost et al (2016), and Iosa et al (2021) all emphasized that poststroke functional outcome is influenced by a combination of stroke severity, neurological impairment, comorbidities, and rehabilitation-related factors.^[12–14]^ Therefore, while the current regression model supports the importance of LOS, LOS should be interpreted as 1 component within a broader recovery framework rather than as an isolated determinant. The finding that the moderate LOS group did not show a statistically significant difference in total-FIM gain after adjustment, despite a significant difference in motor-FIM gain, merits particular attention. This may indicate that the impact of LOS is more domain-specific than global, with motor recovery being more responsive to increased rehabilitation duration than composite total-FIM scores under certain conditions. It is also possible that the moderate LOS group achieved partial functional recovery that was insufficient to produce a significant difference in total-FIM gain once confounding factors were controlled. This partial divergence across outcome measures is consistent with the broader rehabilitation literature, which suggests that recovery domains do not necessarily improve in parallel and may be influenced by different clinical mechanisms.^[15,16]^

In contrast to the findings for total-FIM, motor-FIM, cognitive-FIM gain was not significantly associated with LOS in either unadjusted or adjusted analyses. This result differs somewhat from literature suggesting that cognition is an important determinant of rehabilitation outcome. For example, Cumming et al (2013) described poststroke cognitive deficits as highly prevalent yet incompletely understood in rehabilitation contexts, while Kapoor et al (2019) showed that early cognitive impairment predicts long-term patient-centered outcomes.^[17,18]^ VanGilder et al (2020) also reviewed how poststroke cognitive impairment may influence responsiveness to motor rehabilitation.^[16]^ However, the absence of a significant LOS effect on cognitive-FIM gain in the present study does not necessarily contradict these reports. Rather, it may indicate that cognitive recovery is less directly dependent on LOS alone and may instead be more strongly influenced by baseline cognitive reserve, lesion characteristics, education level, communication deficits, or specific cognitive rehabilitation strategies, as previously suggested.^[19,20]^ Another possible explanation for the nonsignificant cognitive findings is that the cognitive-FIM instrument may be less sensitive to subtle cognitive changes than motor-focused measures are to physical change. Cognitive and communicative impairments after stroke are often heterogeneous and may not improve at the same pace as physical disability. Kaylor and Singh (2023) and Song et al (2024) noted that stroke-related impairments involving speech, language, and swallowing may substantially affect outcomes but can follow distinct recovery trajectories.^[21,22]^ Likewise, Cumming et al (2013) argued that cognitive recovery after stroke remains difficult to characterize comprehensively.^[17]^ Thus, the lack of significant differences in cognitive-FIM gain across LOS groups may reflect both true clinical complexity and measurement limitations.

The current findings should also be interpreted in light of regional and contextual evidence from Saudi Arabia. Al-Jadid and Robert (2010) identified determinants of LOS in an inpatient stroke rehabilitation unit in Saudi Arabia, and Abdul-Sattar and Godab (2013) examined predictors of functional outcome among Saudi stroke patients following inpatient rehabilitation.^[23,24]^ Bindawas et al (2017, 2018) further demonstrated the importance of age and LOS in shaping rehabilitation outcomes within Saudi settings.^[3,25]^ More recently, Aljuhani et al (2024) identified factors influencing stroke recovery and discharge FIM scores in a tertiary hospital.^[26]^ The consistency of the present findings with this national body of literature strengthens the relevance of LOS as a clinically meaningful rehabilitation variable in Saudi Arabia, while also suggesting that local health-system factors, discharge planning practices, and patient case-mix may influence how LOS translates into measurable gain. At the same time, some caution is warranted when interpreting a longer LOS as inherently beneficial. Previous studies have shown that LOS can also be influenced by stroke severity, medical complications, comorbidity burden, and discharge barriers, rather than treatment exposure alone. Bijl et al (2023), Tran et al (2023), and Shao et al (2023) all indicate that LOS may reflect patient complexity as much as rehabilitation opportunity.^[4,27,28]^ Similarly, Simić-Panić (2018), Yang et al (2021), and Alfakeeh et al (2024) highlight the contribution of comorbidities and vascular risk factors to stroke recovery.^[29–31]^ Therefore, although the present regression analysis shows better total and motor gains in the long LOS group, this should not be interpreted simplistically as evidence that extending hospitalization by itself will always improve outcomes. Rather, it is likely that LOS interacts with rehabilitation intensity, baseline severity, complications, and patient-specific recovery potential.

From a quality-of-care perspective, the present findings suggest that a longer inpatient rehabilitation stay may be more efficacious in achieving greater total and motor functional gains after stroke; however, this does not necessarily mean it is always the most efficient approach. While patients with longer LOS demonstrated better rehabilitation outcomes, prolonged hospitalization may also increase healthcare costs, bed occupancy, caregiver burden, and pressure on rehabilitation resources. Previous studies have shown that LOS is influenced not only by therapeutic need, but also by stroke severity, comorbidities, discharge barriers, and health-system factors.^[4,6,23,27]^ Therefore, in the context of healthcare quality, LOS should be interpreted as a balance between effectiveness, efficiency, and resource utilization, with the goal of achieving optimal functional recovery without unnecessary prolongation of inpatient care. These findings highlight the need for individualized rehabilitation planning that maximizes patient benefit while minimizing avoidable system and family burden.

### 4.1. Study limitations

This study had several important limitations. First, many relevant outcome measures, including National Institutes of Health Stroke Scale, Montreal Cognitive Assessment, Mini-Mental State Examination, Timed Up and Go, 10-Meter Walk Test, 6-Meter Walk Test, and Berg Balance Scale, had substantial missing data because they were underused or incompletely documented in medical records. As a result, only FIM and LOS were consistently available for analysis, which limited the evaluation of stroke recovery to FIM-based outcomes. Second, although the study aimed to examine the characteristics of the rehabilitation program, the available records provided only the names of interventions and lacked sufficient detail on their frequency, intensity, duration, progression, protocols, and guidelines. Third, LOS may reflect not only rehabilitation exposure but also other factors such as stroke severity, comorbidities, medical complications, discharge barriers, social factors, and health-system processes; therefore, the observed associations should not be interpreted as strictly causal. Finally, the retrospective study design limited control over data completeness, standardization of outcome measurement, and consistency of rehabilitation documentation.

## 5. Conclusion

This study showed that LOS in inpatient stroke rehabilitation was associated with functional recovery, especially in total-FIM and motor-FIM gain. Patients with a short LOS had significantly lower total-FIM and motor-FIM gains than those with a long LOS, while patients with a moderate LOS also had lower motor-FIM gain. These associations remained significant after adjustment for age, gender, and stroke location. In contrast, cognitive-FIM gain was not significantly associated with LOS. Overall, the findings suggest that a longer inpatient rehabilitation stay may be more beneficial for overall and motor functional recovery after stroke, while cognitive improvement may be influenced by other factors. The results also support the value of inpatient stroke rehabilitation and highlight the need for future prospective studies with broader outcome measures and better documentation of rehabilitation program characteristics.

## Acknowledgments

This study is supported via funding from Prince Sattam bin Abdulaziz University project number (PSAU/2026/R/1447).

## Author contributions

**Conceptualization:** Bashayer A. AlHarbi, Bader Alqahtani.

**Data curation:** Bashayer A. AlHarbi, Faisl AlGarni, Taalaf AlWaqid, Mastourah AlShahrani, Taibah AlShanqiti.

**Formal analysis:** Bashayer A. AlHarbi.

**Funding acquisition:** Bader Alqahtani.

**Investigation:** Bashayer A. AlHarbi, Faisl AlGarni, Bader Alqahtani.

**Methodology:** Bashayer A. AlHarbi, Bader Alqahtani.

**Project administration:** Bashayer A. AlHarbi.

**Resources:** Bashayer A. AlHarbi.

**Software:** Bashayer A. AlHarbi.

**Supervision:** Bashayer A. AlHarbi, Bader Alqahtani.

**Validation:** Bashayer A. AlHarbi, Bader Alqahtani.

**Visualization:** Bashayer A. AlHarbi, Bader Alqahtani.

**Writing—original draft:** Bashayer A. AlHarbi.

**Writing—review & editing:** Bashayer A. AlHarbi.
